# Nomogram-Based Chronic Kidney Disease Prediction Model for Type 1 Diabetes Mellitus Patients Using Routine Pathological Data

**DOI:** 10.3390/jpm12091507

**Published:** 2022-09-14

**Authors:** Nakib Hayat Chowdhury, Mamun Bin Ibne Reaz, Sawal Hamid Md Ali, Shamim Ahmad, María Liz Crespo, Andrés Cicuttin, Fahmida Haque, Ahmad Ashrif A. Bakar, Mohammad Arif Sobhan Bhuiyan

**Affiliations:** 1 Department of Electrical, Electronic and Systems Engineering, Universiti Kebangsaan Malaysia, Bangi 43600, Malaysia; 2 Department of Computer Science and Engineering, Bangladesh Army University of Science and Technology, Saidpur Cantonment, Saidpur 5310, Bangladesh; 3 Department of Computer Science and Engineering, University of Rajshahi, Rajshahi 6205, Bangladesh; 4 Abdus Salam International Centre for Theoretical Physics (ICTP), 34151 Trieste, Italy; 5 Nencki Institute of Experimental Biology, Polish Academy of Sciences, 02-093 Warsaw, Poland; 6 Department of Electrical and Electronics Engineering, Xiamen University Malaysia, Bandar Sunsuria, Sepang 43900, Malaysia

**Keywords:** machine learning, chronic kidney disease, type 1 diabetes mellitus, prediction model, early detection, nomogram

## Abstract

Type 1 diabetes mellitus (T1DM) patients are a significant threat to chronic kidney disease (CKD) development during their life. However, there is always a high chance of delay in CKD detection because CKD can be asymptomatic, and T1DM patients bypass traditional CKD tests during their routine checkups. This study aims to develop and validate a prediction model and nomogram of CKD in T1DM patients using readily available routine checkup data for early CKD detection. This research utilized 1375 T1DM patients’ sixteen years of longitudinal data from multi-center Epidemiology of Diabetes Interventions and Complications (EDIC) clinical trials conducted at 28 sites in the USA and Canada and considered 17 routinely available features. Three feature ranking algorithms, extreme gradient boosting (XGB), random forest (RF), and extremely randomized trees classifier (ERT), were applied to create three feature ranking lists, and logistic regression analyses were performed to develop CKD prediction models using these ranked feature lists to identify the best performing top-ranked features combination. Finally, the most significant features were selected to develop a multivariate logistic regression-based CKD prediction model for T1DM patients. This model was evaluated using sensitivity, specificity, accuracy, precision, and F1 score on train and test data. A nomogram of the final model was further generated for easy application in clinical practices. Hypertension, duration of diabetes, drinking habit, triglycerides, ACE inhibitors, low-density lipoprotein (LDL) cholesterol, age, and smoking habit were the top-8 features ranked by the XGB model and identified as the most important features for predicting CKD in T1DM patients. These eight features were selected to develop the final prediction model using multivariate logistic regression, which showed 90.04% and 88.59% accuracy in internal and test data validation. The proposed model showed excellent performance and can be used for CKD identification in T1DM patients during routine checkups.

## 1. Introduction

According to the 10th Diabetes Atlas, 2021, by the International Diabetic Federation (IDF), around 537 million adults (20–79 years old) are suffering from diabetes mellitus (DM), and by 2030, this number is expected to rise to 643 million [1]. One-tenth of these diabetes patients have type 1 diabetes mellitus (T1DM), and an additional 1.2 million children and teenagers under the age of 20 also have type 1 diabetes [1]. Chronic kidney disease (CKD) is one of the severe consequences of T1DM, and nearly half of all T1DM patients have a chance of developing CKD at some point in their lives [2]. Diabetic kidney disease is a primary cause of the end-stage renal disease (ESRD) in North America [3]. An ESRD patient needs regular dialysis or kidney transplantation to survive. Healthcare and medication costs rise as CKD progresses, and there is a greater risk of additional adverse effects, including increased risk of death, progression to end-stage kidney disease, and increased risk of diseases of the heart and arteries [4]. In the United States, kidney diseases are the leading cause of death [5].

According to the KDIGO (Kidney Disease Improving Global Outcomes) guidelines, CKD is defined by having an estimated glomerular filtration rate (eGFR) value of less than 60 mL/min/1.73 m^2^ for more than three months [6]. CKD symptoms include fatigue, fluid retention, anomalies in the urine, nausea, vomiting, and neurological and cognitive impairment [7]. However, it is asymptomatic in most cases; 90% of CKD patients do not know they have CKD [5]. As a result, there is always a chance of delay in identifying CKD in T1DM patients. On the other hand, early detection of CKD can assist patients in receiving effective medications and interventions that can postpone the loss of renal function or even reverse moderate impairment [8]. Therefore, a precise CKD prediction model based on routinely available data can be useful in identifying individuals who are at higher risk of renal function decline and may benefit from more intensive care.

Machine learning (ML) approaches have recently shown great promise in disease detection and prediction [9,10,11,12,13]. These algorithms can help clinicians make better decisions by extracting information from incomplete, complicated data. Here, incomplete data means data with missing values, and clinical data often suffer from missing values. A dataset can be considered complex data if it has many features (maybe very few of these features are essential for a proposed task), has a complex structure (it is common for clinical data from multiple sources, or even separate tables from the same source, to refer to the same information but have completely distinct structures), or has a vast amount of data. As clinical decision support systems, researchers are already developing machine learning-based prediction models based on clinical data, with better performance than traditional methodologies [14,15,16]. Therefore, to develop a CKD prediction model for T1DM patients, the use of an ML model can be a viable option.

Although there are several machine learning-based CKD prediction models, some did not emphasize diabetic patients [7,17], whereas others only considered type 2 diabetes patients while developing their models [18,19,20,21,22,23]. However, type 1 diabetes is different from type 2 diabetes [24,25]. Type 1 diabetes is a genetic condition that often manifests early in life, whereas type 2 diabetes is primarily lifestyle-related and develops over time, with the genes involved remaining unknown. People with type 1 diabetes commonly have HLA-DR3 or HLA-DR4 genes, which are linked to autoimmune disease. The patient’s immune system attacks and destroys insulin-producing cells in the pancreas in type 1 diabetes. As a result, T1DM patients can no longer produce insulin and must rely on daily insulin injections to control their blood glucose levels. Type 2 diabetes, on the other hand, is known as insulin resistance. In this case, either the patient’s body does not produce enough insulin, or the patient’s insulin does not function properly. In addition, type 2 diabetes is strongly linked to lifestyle, obesity, family history, and ethnicity. Type 1 diabetes, on the other hand, is unaffected by lifestyle or weight, and patients cannot reduce their risk of developing type 1 diabetes through lifestyle changes. T1DM is most common in young adults [26]. Patients with type 1 diabetes are more likely to be exposed to diabetes-related risk factors over a more extended period than type 2 diabetes. Moreover, the genetic risk factors behind diabetes causally increase the risk of CKD in T1DM [27]. As a result, adult T1DM patients are more likely than T2DM individuals to develop CKD and ESKD [28].

Only two studies designed kidney disease prediction models for type 1 diabetes patients [29,30], one of which employed Poisson regression to create an End-Stage Kidney Disease (ESKD) prediction model [29]. Other research developed a renal disease development prediction model using ridge regression [30]. Both models used urinary albumin (urine protein) as an essential variable, but diabetes patients tend to bypass this test. Only half of the USA diabetic patients conduct tests for urine albumin [3]. Moreover, none of these models focused on the early detection of kidney disease.

This study aimed to develop and validate a CKD classification model and nomogram using multivariate linear regression. The objective was to develop and validate a prediction model for T1DM patients that could be operated on readily available patients’ routine checkup data to identify asymptomatic CKD at the earliest possible time.

## 2. Materials and Methods

**Figure 1:**
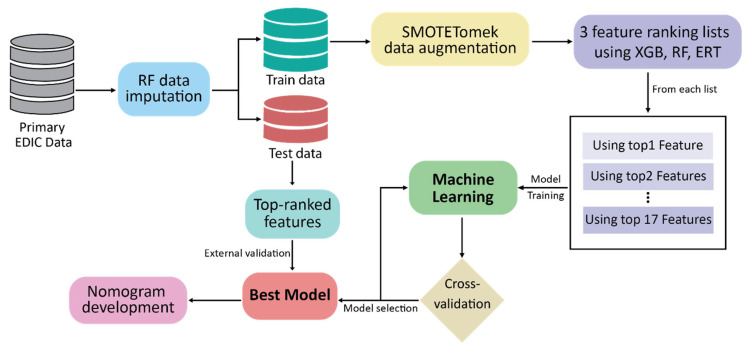
Overall working procedure.

Figure 1 illustrates the overall working framework of this study. The following sections describe the processes in detail.

### 2.1. Dataset Description

The GFR dataset from the Epidemiology of Diabetes Interventions and Complications (EDIC) clinical trial was used in this research. The EDIC study was carried out by the National Institute of Diabetic, Digestive and Kidney Diseases (Bethesda, MD, Montgomery, MD, USA) to see how rigorous diabetes medication affected T1DM patients [31,32]. In 1994, 1375 T1DM patients were enrolled in the EDIC trial at 28 sites in the USA and Canada, which is still ongoing. EDIC is a longitudinal study in which the patients’ ages ranged from 19 to 57 at the start, and the GFR dataset contains 16 years of EDIC trial information; thus, the dataset had information on patients ranging from 19 to 73 years old. In the EDIC study, patients’ demographic, behavioral, and medication usage data were collected by self-report. Each clinical parameter measurement was performed utilizing standardized methodologies in the EDIC central biochemistry laboratory, and measurement drift was prevented using long-term quality control procedures [32,33].

This study included all patients aged eighteen years old or above with a clinical diagnosis of T1DM. The baseline date was defined as the data from the EDIC trial’s first year. We excluded all samples having a missing value in the output column. We also excluded duplicate samples. This study evaluated 1375 participants’ 16 years of data and finally selected 1752 samples after removing all duplicates to create the primary dataset.

This study included 17 demographics, disease characteristics, medication history, and clinical parameters that are easily accessible through routine checkups of a T1DM patient. Selected predictors were age, sex, body mass index (BMI), smoking and drinking habit, hypertension, use of ACE inhibitors, hypercholesterolemia, duration of insulin-dependent diabetes mellitus (IDDM), glycated hemoglobin (HbA1c) levels, total cholesterol, triglycerides, high-density lipoproteins (HDL), low-density lipoproteins (LDL), systolic blood pressure (SBP), diastolic blood pressure (DBP), and mean blood pressure (mean BP). In several previous investigations, these factors were considered to be crucial for CKD identification [7,17,21,34,35,36]. The study had two outcomes: CKD and non-CKD, where the CKD class indicated that the participant had chronic kidney disease and the non-CKD class indicated otherwise.

In the EDIC study, experts evaluated participants’ BMI, HbA1c levels, and blood pressure on an annual basis [33]. Incident hypertension was defined as a systolic blood pressure of 140 mmHg and/or diastolic blood pressure of 90 mmHg on two successive annual appointments [33]. The albumin excretion rate (AER) and fasting lipid levels (cholesterol, triglycerides, HDL, and LDL) were assessed every two years. Serum creatinine levels were measured annually at the EDIC Central Biochemistry Laboratory, University of Minnesota [37]. Estimated GFR (eGFR) was calculated using data on blood creatinine levels, age, sex, and race using the Chronic Kidney Disease Epidemiology Collaboration (CKD-EPI) method [31,38]. CKD was defined as an eGFR of less than 60 mL/min/1.73 m^2^ for at least two consecutive collections.

### 2.2. Data Preprocessing and Feature Ranking

There were 33 missing values in four variables of the primary dataset: smoking and drinking habits, usage of ACE medications, and hypertension. In this research, the random forest (RF) data imputation technique was used to fill all missing data, as this technique showed better performance than other techniques for medical data imputation [39].

After addressing the missing values, the primary dataset was partitioned into 80% training and 20% testing datasets. The prediction model was trained using the training dataset only, and the testing dataset was kept as it was and used for further validation to evaluate the selected model’s performance on unseen data. The dataset was imbalanced; among the 1754 samples, 391 had CKD. So, the training dataset was balanced using the SMOTE-Tomek data augmentation technique [40] before training the classifier model. The SMOTE-Tomek approach combines the Synthetic Minority Oversampling Technique (SMOTE) [41] and Tomek Links [42] under-sampling technique.

This study implemented three feature ranking techniques: extreme gradient boosting (XGB) [43], random forest (RF) [44], and extremely randomized trees (ERT) [45] on the training dataset to identify feature importance. Initially, all 17 features were assessed using these techniques to identify the top predictors of CKD in T1DM patients. Then, features were ranked based on their relative importance in predicting CKD. Thus, three separate feature ranking lists were created, and after evaluating these lists, the best performing list was selected. Data imputation, data augmentation, and feature ranking were performed using the in-house build Python 3.8 code.

### 2.3. Statistical Analyses

In order to check the normality of the different features, the Shapiro–Wilk test was performed with a *p*-value of 0.05. None of the variables followed the Gaussian distribution. Levene’s test was performed with a *p*-value of 0.05 to test the homogeneity of variance for both CKD and non-CKD groups. Ten features (age, drinking, use of ACE inhibitors, hypercholesterolemia, BMI, SBP, DBP, total cholesterol, HDL, LDL) show a homogeneity of variance for both groups. In-house Python 3.8 code, scipy and pingouin machine learning library were used for these tests. For baseline characteristics, quantitative data are reported as means ± standard deviation, maximum and minimum value, whereas qualitative variables are presented as frequency and percentage (%). The number of missing values was also reported for each variable. In-house build Python 3.8 code and scikit-learn were used for statistical analysis. Scikit-learn is a free software machine learning library for the Python programming language.

### 2.4. Development of Prediction Model

This study applied multivariate logistic regression (LR) to develop the CKD prediction model. In order to achieve the best performance using a minimum number of predictors, top-ranked feature combinations from all three feature raking techniques were used separately to develop different LR models. This study used the top-1 feature, then top-2 features, top-3 features, and so on until the top-17 features to develop 17 different models for every feature ranking list. Each model’s performance was evaluated using stratified 10-fold cross-validation to select the best performing model with minimum features. An in-house-built Python 3.8 code was used to develop all LR models. The top-ranked features were used to create the multivariate logistic regression-based prediction model through Stata/MP software (version v13.0, StataCorp LLC, College Station, TX, USA), which was used to develop the CKD probability prediction equation for T1DM patients.

### 2.5. Validation of CKD Prediction Models

Calibration curves for internal validation (using train dataset) and test data validation were generated to assess the model’s goodness of fit. The calibration is considered good if the calibration line between the projected probability and the observed outcome matches the ideal standard line. The decision curve analysis (DCA) was utilized to assess the selected model’s clinical utility and depict its potential net benefit. The threshold values were determined using decision curve analysis (DCA) for each variable individually and all variables together. The Fisher exact probability test was also performed to determine the relationship between the predicted and the actual result.

### 2.6. Development of the Nomogram

For easy application in routine clinical practice, a nomogram was generated from the multivariable logistic regression model using the Stata nomolog package developed by Zlotnik et al. [46]. Only the final model’s predictors were used in nomogram development.

## 3. Results

### 3.1. Baseline Characteristics

Table 1 shows the baseline characteristics of EDIC patients. In total, 1375 T1DM patients were enrolled in EDIC studies. The average age of the EDIC patients in the first year was 35.12 ± 6.97 years (657 female and 718 male), and the mean diabetic duration was 13.64 ± 4.94 years.

At the beginning of the EDIC study, only four participants had CKD, and during the 16 years of the EDIC study, 66 more participants were diagnosed with CKD. This study considered 16 years of data of EDIC study and primarily selected 1752 samples, and 391 of these samples had CKD. Then, the primary data were divided into two parts, 80% data as a training set and 20% as a test set. After performing the SMOTETomek data augmentation technique on the training dataset, it had a total of 2710 samples, where 1361 were CKD samples and 1349 were non-CKD samples.

### 3.2. Performance Analysis of the Feature Ranking Techniques

**Figure 2:**
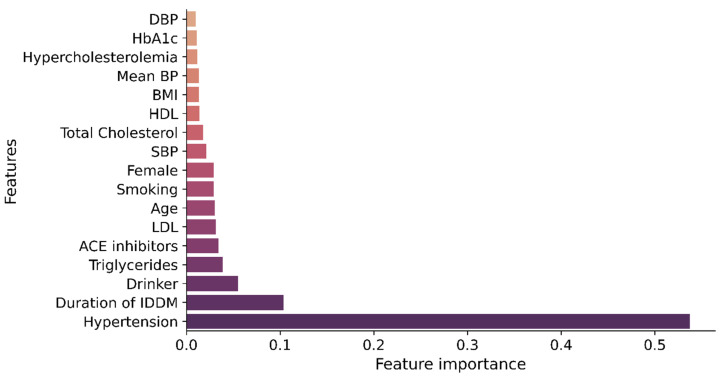
Feature ranking using XGB algorithm.

This study applied three feature-ranking techniques (XGB, RF, ERT) to the training dataset, creating three feature ranking lists. In all three lists, hypertension and duration of IDDM were the top-2 ranked features. Triglycerides, ACE inhibitors, drinking, and age were also found as significant variables in these lists. However, their relative importance and position were different on different lists. Figure 2 depicts the relative relevance of features created by the XGB algorithms on the training dataset. The relative relevance of features created by the RF and ERT algorithms can be found in Supplementary Figures S1 and S2.

**Figure 3:**
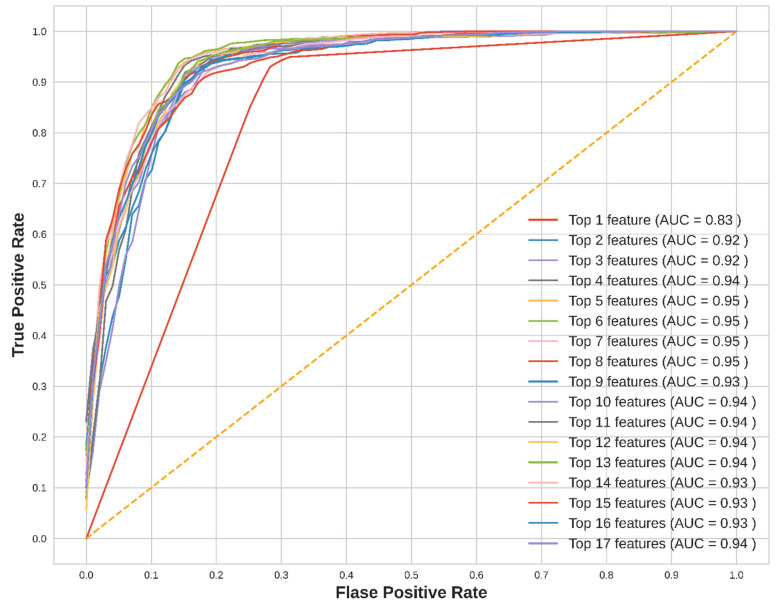
The ROC curves for the top 1 to top 17 ranked features using XGB feature ranking and LR classifier.

Receiver operating characteristic (ROC) curves were generated using LR classifier for each ranked features list to understand their impact on CKD prediction. Figure 3 shows the ROC curves for the XGB feature ranking list using the top 1 to top 17 features. The ROC curves plot for the RF and ERT feature ranking lists are given in the Supplementary Figures S3 and S4. It can be observed from Figures S2–S4 that after the top 5 features, the areas under the curve (AUC) became saturated at 95% for all other features added to the lists.

In order to find the best combination of top-ranked features for identifying CKD patients, logistic regression classifiers were used to evaluate the performance of the features by using the top-1 features, top-2 features, and up to top-17 features in an incremental manner for all three feature ranking techniques. Ten-fold cross-validation was used to train and test the LR models, and each model’s performance was evaluated based on the sensitivity (recall), specificity, accuracy, precision, and F1 values. As shown in Table 2, the top-8 ranked features from the XGB feature ranking technique achieved the best performance with 90% accuracy in 10-fold cross-validation, and the ranked variables were hypertension, duration of IDDM, drinking, triglycerides, ACE inhibitors, LDL, age, and smoking. The RF and ERT feature ranking list achieved a maximum of 89% accuracy, and details of every model using the RF and ERT feature ranking techniques can be found in Supplementary Tables S1 and S2.

### 3.3. CKD Prediction Model

The final prediction model was created using multivariate logistic regression utilizing the top-8 features ranked by the XGB feature ranking approach. Table 3 shows the regression coefficient of each predictor, z-value, standard error, statistical significance, and the 95% confidence interval. The z-values show which factors are important in detecting CKD. The greater the value, the greater the feature’s contribution to CKD prediction. According to the z-values of these parameters, hypertension, IDDM duration, and triglycerides are substantial factors, as there had a greater z-value range from 16.83 to 11.07. However, all variables had a *p*-value of less than 0.05, suggesting a significant link to CKD identification.

The CKD prediction equation was developed by adding the product of coefficients and the values of each parameter with the model constant, as shown in Equation (1). Then, the value of Equation (1) was used in the sigmoid function to generate the probability (Equation (2)). The threshold value for Equation (2) was 0.5, and patients with more than 0.5 values from Equation (2) were predicted as CKD patients.

(1) $$\begin{array}{cc} LP=-11.39143 & +\left(3.070858\times Hypertension\right)+\left(0.2398436\times Duration\;of\;IDDM\right)\\ & +\left(-1.486273\times Drinking\right)+\left(0.0125956\times Triglycerides\right)\\ & +\left(0.5133911\times ACE\;inhibitors\right)+\left(-0.0071267\times LDL\right)+\left(0.0923286\times Age\right)\\ & +\left(-1.185757\times Smoking\right) \end{array}$$

(2) $$CKD\;Probability=\frac{1}{1+e^{-\left(LP\right)}}$$

Table 4 shows the performance assessment metrics for the CKD prediction model in training and testing datasets, and the model had 90.04% accuracy in train data and 88.59% accuracy in test data. Among the 1349 non-CKD T1DM patients for the training dataset, 87.47% were identified successfully as non-CKD patients, and 12.53% were misclassified as CKD patients. Whereas for the 1361 CKD patients, 92.06% were appropriately classified as CKD patients, and 7.9% were misclassified as non-CKD patients. On the other hand, for the test dataset, for the 394 non-CKD class participants, 88.58% were classified accurately as non-CKD patients and 12.42% were classified inaccurately as CKD. In addition, among 132 CKD patients, 90.91% were identified successfully as CKD and 9.09% were identified wrongly as non-CKD. Table 5 represents the Fisher exact probability test to determine the relationship between the predicted CKD and non-CKD classes and the actual result for the train and test dataset.

**Figure 4:**
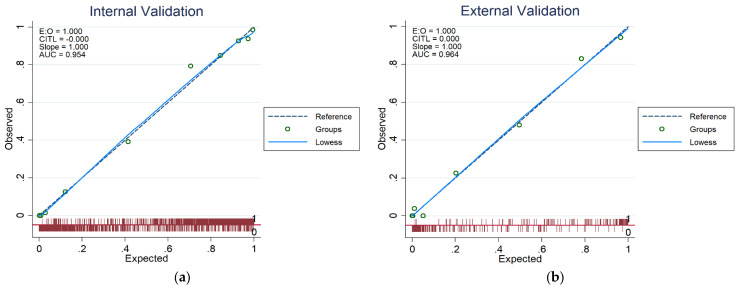
Calibration graphs comparing calculated CKD probability and actual value for (**a**) the train set (internal) validation, (**b**) the test data validation.

**Figure 5:**
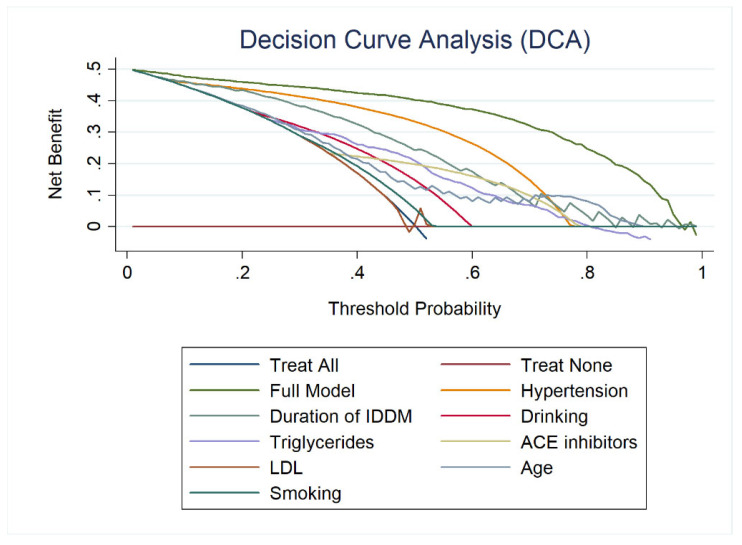
Decision curve analysis (DCA) of the top 8 ranked features for predicting the CKD.

**Figure 6:**
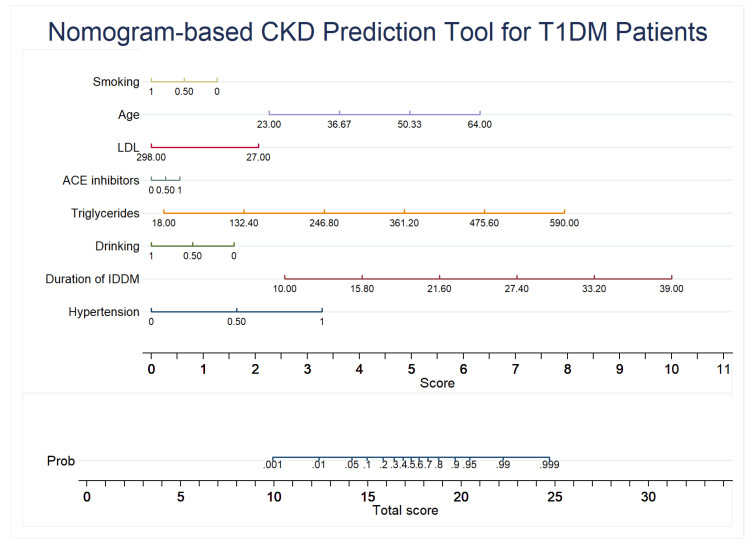
Nomogram to predict the likelihood of CKD in T1DM patients.

A calibration plot was created to test the model’s dependability. As demonstrated in Figure 4, the calibration graphs for both internal (train set) and test data validation were quite near the diagonal line, indicating the model’s dependability. Figure 5 shows that all predictors have a positive net benefit threshold for CKD detection, with the top-8 rated features’ model reaching a threshold greater than 0.95. Finally, a multivariate logistic regression-based nomogram was generated using these eight features, as presented in Figure 6.

## 4. Discussion

CKD is asymptomatic in most cases, and 90% of CKD patients have no idea that they have CKD [5]. So, there is always a chance of delay in detecting CKD. However, early detection of CKD can help the patients receive intensive care that can delay or even stop loss in renal function. Although around 50% of type 1 diabetes mellitus (T1DM) patients have a risk of developing chronic kidney disease (CKD) in their lifetime [2], unfortunately, there has been very little research to detect CKD in people with type 1 diabetes compared to type 2 diabetes. Nonetheless, there is a distinction between type 1 and type 2 diabetes. Type 2 diabetes is primarily a lifestyle disease, whereas type 1 diabetes is a genetic disorder. Furthermore, the genetic risk factors for diabetes increase the risk of CKD in T1DM [27]. As a result, adults with T1DM are more prone to develop CKD and ESKD than T2DM patients [28].

In one study, Vistisen et al. [29] applied Poisson regression to build an End-Stage Kidney Disease (ESKD) identification model for the T1DM population, with C-statistics ranging from 0.88 to 0.96. In another study, Colombo et al. [30] applied ridge regression to construct a prediction model for T1DM patients’ kidney disease progression. Both studies used albuminuria, serum creatinine, and current GFR as important predictors. However, these parameters are kidney damage markers, and general T1DM patients or asymptomatic CKD patients may not have these tests during their regular check-ups. In these cases, the above-discussed models could not be effective.

This study utilized 16 years of longitudinal data from the EDIC study to build a CKD prediction model for T1DM patients that can be operated on using simple routine checkup data. This research considered 17 parameters: age, sex, BMI, smoking and drinking habit, hypertension, use of ACE inhibitors, hypercholesterolemia, duration of insulin-dependent diabetes mellitus (IDDM), glycated hemoglobin (HbA1c) levels, total cholesterol, triglycerides, high-density lipoproteins (HDL), low-density lipoproteins (LDL), systolic blood pressure (SBP), diastolic blood pressure (DBP), and mean blood pressure (mean BP). These variables are readily available from T1DM patients’ self-assessments and routine checkups.

Datasets from clinical trials always suffer from missing entries and unbalance among different patient classes. The EDIC dataset also had missing entries in several features and imbalance among CKD and non-CKD classes. The missing values were imputed using the RF imputation technique. Then, the imputed dataset was divided into 80% train and 20% test datasets. The train dataset was used for model’s training and internal validation, where the test dataset was used to test the developed model. To balance the dataset, a combination of oversampling and under-sampling techniques, SMOTETomek [40], was applied to the training dataset.

Then, three different feature ranking techniques, extreme gradient boosting (XGB), random forest (RF), and extremely randomized trees (ERT), were used to create three ranked lists of features based on their relative importance on CKD prediction. The logistic regression (LR) classifier was used to find out the best-performing feature combinations from these ranked feature lists. Top-8 variables using XGB feature ranking provided the best performance for the LR classifier. This study found hypertension, duration of IDDM, drinking, triglycerides, ACE inhibitors, LDL, age, and smoking habits as the most significant predictors for CKD prediction in T1DM patients. A multivariable logistic regression model was then used with these top-8 features to develop a CKD prediction model for T1DM patients. A diagnostic nomogram was created to conveniently implement the prediction model in T1DM patients’ routine clinical practice.

The proposed model showed reliable performance with 90.04% and 88.59% accuracy on the train and test datasets. In addition, the developed model showed 90–90% accuracy in identifying CKD and 87–88% accuracy in non-CKD patients in train and test data.

As per our knowledge, this is the first study that used a logistic regression-based nomogram to generate a CKD prediction model, which was solely developed for the T1DM population. The strength of this study is that it was based on a large cohort of patients from 28 different medical institutions that took part in EDIC trials, offering a broad collection of variables to the created model. Another significant advantage of the study is that the developed model uses simple data available from self-assessment and routine patient follow-up to provide a reliable result without any delay. As a result, this model can be a valid alternative to predict CKD when complex laboratory tests are unavailable. Furthermore, this model can be used as an assisting tool during T1DM patients’ routine visits to make an educated guess regarding their CKD status, thereby improving the odds of detecting asymptomatic CKD patients early.

This study has some limitations. This study prepared the test dataset from the same cohort, and external validation of the proposed model in other cohorts may prove the model’s robustness. This model did not consider patients’ family history of CKD, daily lifestyle, or food habits, as these are not part of routine medical checkups, but these could be valuable predictors.

## 5. Conclusions

In this research, a CKD prediction model was developed for T1DM patients using multivariate logistic regression and readily available features with 90.04% accuracy in internal validation and 88.59% accuracy in test data validation. This study has also produced CKD prediction equations and a nomogram for T1DM patients that can be utilized as a secondary decision support system for health professionals to identify CKD in T1DM patients during routine checkups, improving their healthcare.

## Figures and Tables

**Table 1:** Baseline Characteristics of the EDIC Patients.

N = 1375	Missing Values	Min	Max	Mean (±Sd)
Age (years)	0	19.00	57.00	35.12 (±6.97)
Sex, n (%)	0			
Male	718 (52%)			
Female	657 (48%)			
BMI (kg/m^2^)	0	16.62	66.01	26.10 (±4.07)
Diabetic Duration (years)	0	6.00	28.00	13.62 (±4.95)
ACE Inhibitors, n (%)	0	91 (6%)		
Hba1c (%)	4	4.40	15.10	8.14 (±1.39)
HDL Cholesterol (mg/dL)		25.00	103.00	52.54 (±13.10)
LDL Cholesterol (mg/dL)	0	26.00	310.00	113.94 (±30.57)
Total Cholesterol (mg/dL)	0	85.00	444.00	183.59 (±35.97)
Hypertension, n (%)	8	232 (16%)		
Hypercholesterolemia, n (%)	0	397 (28%)		
Triglycerides (mg/dL)	0	17.00	1110.00	86.44 (±64.43)
Systolic BP (mmHg)	0	82.00	172.00	117.41 (±12.64)
Diastolic BP (mmHg)	0	40.00	116.00	75.00 (±9.30)
Mean BP (mmHg)	0	59.33	134.00	89.13 (±9.40)
Smoking, n (%)	8	274 (19%)		
Drinking, n (%)	13	485 (35%)		

Abbreviations: BMI, body mass index; ACE, angiotensin-converting enzyme; Hba1c, glycated hemoglobin; HDL, high-density lipoproteins; LDL, low-density lipoproteins (LDL); BP, blood pressure.

**Table 2:** Performance analysis of LR models using top-1 to top-17 features from XGB feature ranking technique.

	Sensitivity	Specificity	Accuracy	Precision	Recall	F1 Score	Non-CKD		CKD	
							TN	FP	FN	TP
Top-1 Feature	0.95 (±0.03)	0.72 (±0.04)	0.83 (±0.02)	0.77 (±0.02)	0.95 (±0.03)	0.85 (±0.01)	972	382	70	1291
Top-2 Features	0.92 (±0.04)	0.84 (±0.04)	0.88 (±0.03)	0.85 (±0.03)	0.92 (±0.04)	0.88 (±0.03)	1137	217	111	1250
Top-3 Features	0.91 (±0.06)	0.85 (±0.02)	0.88 (±0.03)	0.86 (±0.02)	0.91 (±0.06)	0.89 (±0.03)	1157	197	121	1240
Top-4 Features	0.92 (±0.06)	0.86 (±0.03)	0.89 (±0.03)	0.87 (±0.03)	0.92 (±0.06)	0.89 (±0.03)	1170	184	113	1248
Top-5 Features	0.93 (±0.05)	0.86 (±0.02)	0.89 (±0.03)	0.87 (±0.02)	0.93 (±0.05)	0.90 (±0.03)	1167	187	102	1259
Top-6 Features	0.92 (±0.06)	0.86 (±0.03)	0.89 (±0.03)	0.87 (±0.02)	0.92 (±0.06)	0.89 (±0.04)	1168	186	109	1252
Top-7 Features	0.93 (±0.04)	0.86 (±0.02)	0.89 (±0.02)	0.87 (±0.01)	0.93 (±0.04)	0.90 (±0.02)	1159	195	98	1263
**Top-8 Features**	**0.92 (±0.07)**	**0.87 (±0.04)**	**0.90 (±0.04)**	**0.88 (±0.03)**	**0.92 (±0.07)**	**0.90 (±0.04)**	**1176**	**178**	**107**	**1254**
Top-9 Features	0.91 (±0.06)	0.84 (±0.03)	0.88 (±0.03)	0.85 (±0.03)	0.91 (±0.06)	0.88 (±0.03)	1137	217	116	1245
Top-10 Features	0.92 (±0.08)	0.86 (±0.03)	0.89 (±0.04)	0.87 (±0.03)	0.92 (±0.08)	0.89 (±0.04)	1160	194	114	1247
Top-11 Features	0.90 (±0.07)	0.85 (±0.04)	0.88 (±0.03)	0.86 (±0.03)	0.90 (±0.07)	0.88 (±0.03)	1148	206	130	1231
Top-12 Features	0.91 (±0.06)	0.85 (±0.03)	0.88 (±0.02)	0.86 (±0.02)	0.91 (±0.06)	0.88 (±0.02)	1149	205	122	1239
Top-13 Features	0.92 (±0.04)	0.85 (±0.03)	0.88 (±0.01)	0.86 (±0.02)	0.92 (±0.04)	0.89 (±0.01)	1147	207	113	1248
Top-14 Features	0.91 (±0.05)	0.86 (±0.03)	0.89 (±0.03)	0.87 (±0.02)	0.91 (±0.05)	0.89 (±0.03)	1171	183	117	1244
Top-15 Features	0.91 (±0.03)	0.86 (±0.02)	0.88 (±0.02)	0.87 (±0.02)	0.91 (±0.03)	0.89 (±0.02)	1163	191	122	1239
Top-16 Features	0.90 (±0.07)	0.85 (±0.05)	0.87 (±0.05)	0.86 (±0.05)	0.90 (±0.07)	0.88 (±0.05)	1150	204	137	1224
Top-17 Features	0.91 (±0.04)	0.86 (±0.04)	0.89 (±0.04)	0.87 (±0.03)	0.91 (±0.04)	0.89 (±0.04)	1165	189	116	1245

Abbreviations: TN, true negative; FP, false positive; FN, false negative; TP, true positive.

**Table 3:** Using the XGB feature ranking approach, the multivariate logistic regression analysis for CKD prediction with the top-8 factors.

Features	Coef.	Std. Err.	z	*p* > z	(95% Conf. Interval)	
					Lower Limit	Upper Limit
Hypertension	3.070858	0.1824329	16.83	0.000	2.713296	3.42842
Duration of IDDM	0.2398436	0.0162	14.81	0.000	0.2080922	0.271595
Drinking	−1.486273	0.1685275	−8.82	0.000	−1.816581	−1.155966
Triglycerides	0.0125956	0.0011376	11.07	0.000	0.0103659	0.0148252
ACE inhibitors	0.5133911	0.1522363	3.37	0.001	0.2150134	0.8117687
LDL	−0.0071267	0.0018498	−3.85	0.000	−0.0107523	−0.0035011
Age	0.0923286	0.0094812	9.74	0.000	0.0737458	0.1109114
Smoking	−1.185757	0.2162176	−5.48	0.000	−1.609535	−0.7619781
_cons	−11.39143	0.633072	−17.69	0.000	−12.63223	−10.15064

**Table 4:** Performance analysis of the final model on the train and test dataset.

	Sensitivity (%)	Specificity (%)	Accuracy (%)	Precision (%)	F1 Score (%)	Confusion Matrix			
						Non-CKD		CKD	
						TN	FP	FN	TP
EDIC Train Set	92.95	87.10	90.04	87.91	90.36	1175	174	96	1265
EDIC Test Set	91.67	87.56	88.59	71.18	80.13	345	49	11	121

**Table 5:** The Fisher exact probability test for the train and test dataset.

	Train Dataset	Predicted Outcome (Train Dataset)		
		Non-CKD	CKD	Total
Actual Output	Non-CKD (1349)	1175 (87.10%)	174 (12.90%)	1349
	CKD (1361)	96 (7.05%)	1265 (92.95%)	1361
	Total (2710)	1271	1439	2710
	Test dataset	Predicted Outcome (Test dataset)		
		Non-CKD	CKD	Total
	Non-CKD (394)	345 (87.56%)	49 (12.44%)	394
	CKD (132)	11 (8.33%)	121 (91.67%)	132
	Total (526)	356	170	526

## Data Availability

Restrictions apply to the availability of these data. Data were obtained from the National Institute of Diabetes and Digestive and Kidney Diseases (NIDDK) (Bethesda, MD, USA) and are available (https://repository.niddk.nih.gov/studies/edic/, accessed on 6 January 2022) with the permission of NIDDK.

## Supplementary Materials

The following supporting information can be downloaded at: https://www.mdpi.com/article/10.3390/jpm12091507/s1, Supplementary Figure S1: Feature ranking list using RF algorithm; Supplementary Figure S2: Feature ranking list using ERT algorithm; Supplementary Figure S3: The ROC curves for the top-1 to top-17 ranked features using RF feature ranking and LR classifier; Supplementary Figure S4: The ROC curves for the top-1 to top-17 ranked features using ERT feature ranking and LR classifier; Supplementary Table S1. Performance analysis of LR models using top 1 to top 17 features from RF feature ranking technique; Supplementary Table S2. Performance analysis of LR models using top 1 to top 17 features from ERT feature ranking technique.
