# Supplementary material for: Nomogram-Based Chronic Kidney Disease Prediction Model for Type 1 Diabetes Mellitus Patients Using Routine Pathological Data
Source: J Pers Med. 2022 Sep 14;12(9):1507. doi: 10.3390/jpm12091507 (PMC9501949; doi:10.3390/jpm12091507)
Supplement: Supplementary file 1 [file jpm-12-01507-s001.zip › jpm-1897486-supplementary.pdf]

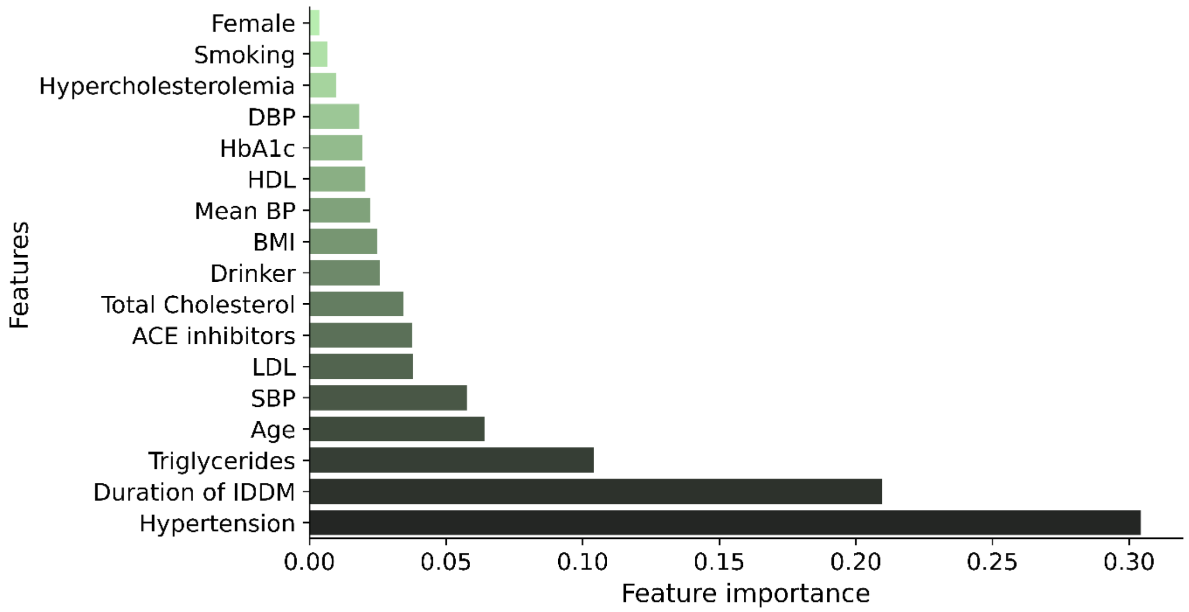

Supplementary Figure S1. Feature ranking list using RF algorithm.

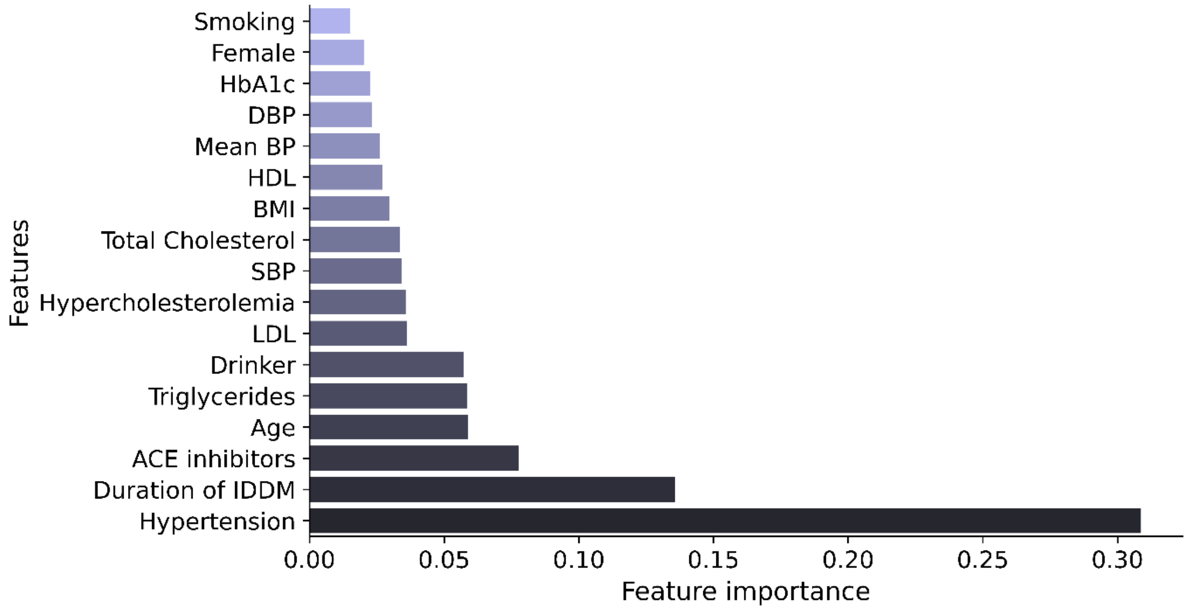

Supplementary Figure S2: Feature ranking list using ERT algorithm.

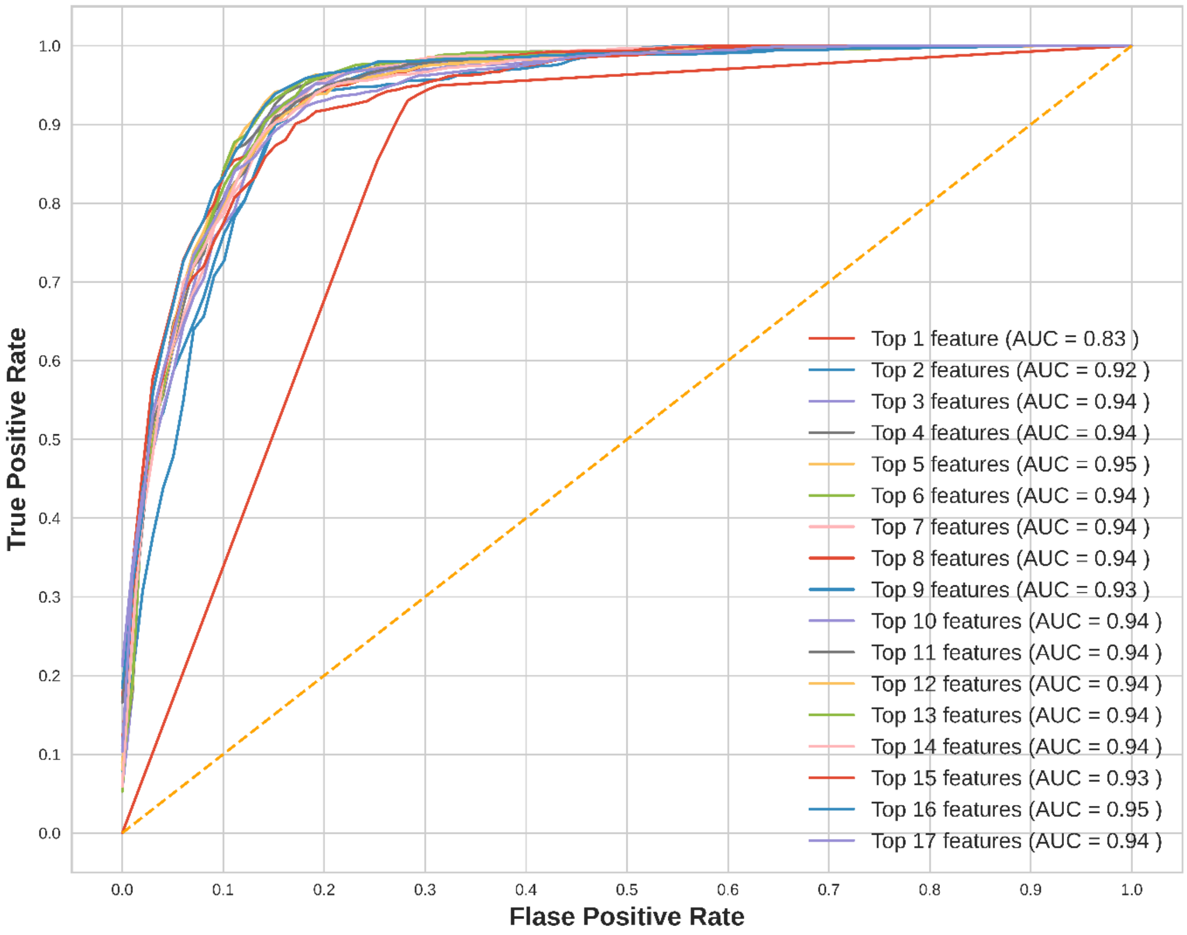

Supplementary Figure S3. The ROC curves for the top 1 to top 17 ranked features using RF feature ranking and LR classifier.

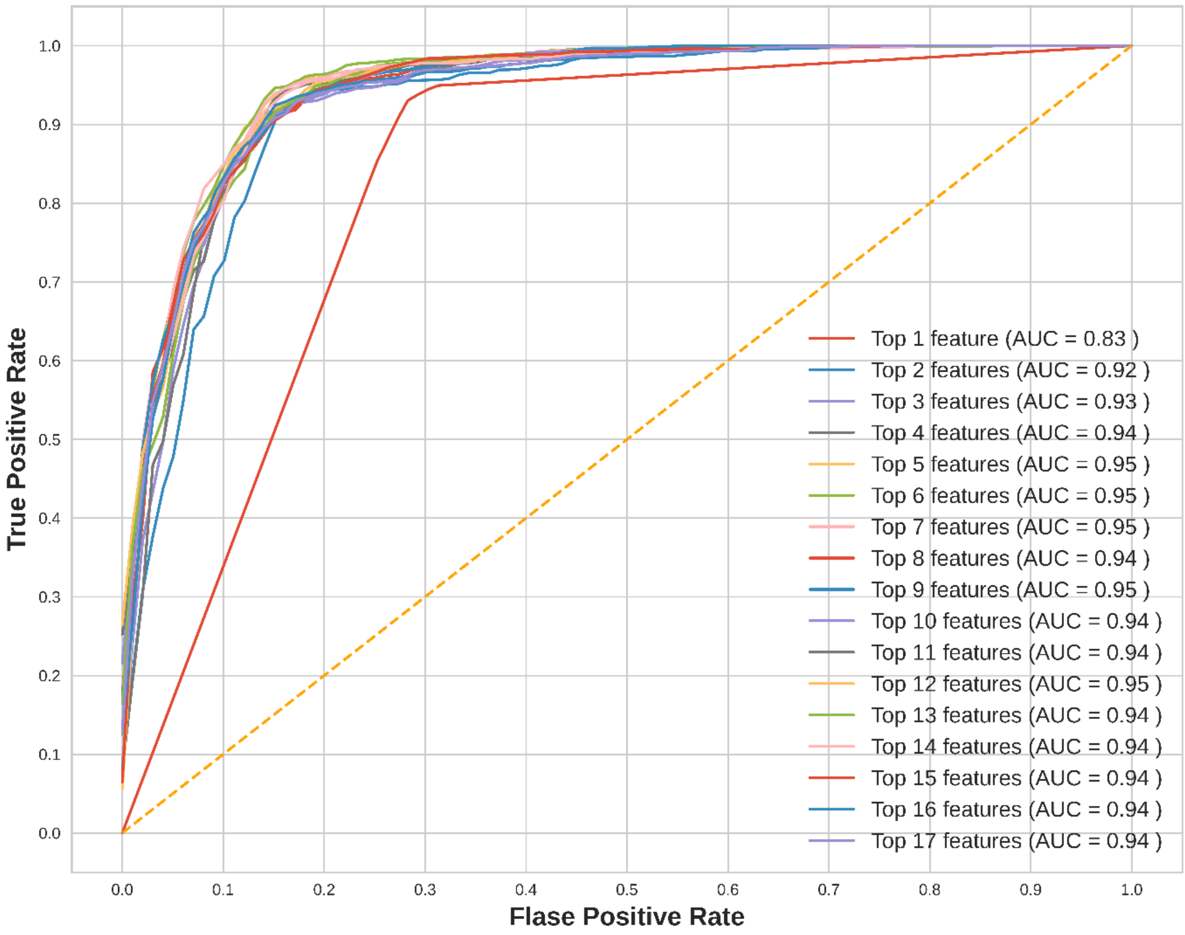

Supplementary Figure S4. The ROC curves for the top 1 to top 17 ranked features using ERT feature ranking and LR classifier.

Supplementary Table S1. Performance analysis of LR models using top 1 to top 17 features from RF feature ranking technique.

	**Sensitivity**	**Specificity**	**Accuracy**	**Precision**	**Recall**	**F1 Score**	**Non-CKD**		**CKD**	
							**TN**	**FP**	**FN**	**TP**
Top-1 Feature	0.95(+/−0.02)	0.72(+/−0.03)	0.83(+/−0.02)	0.77(+/−0.02)	0.95(+/−0.02)	0.85(+/−0.01)	974	379	72	1289
Top-2 Features	0.93(+/−0.04)	0.84(+/−0.03)	0.88(+/−0.03)	0.85(+/−0.02)	0.93(+/−0.04)	0.89(+/−0.03)	1134	219	101	1260
Top-3 Features	0.93(+/−0.05)	0.85(+/−0.03)	0.89(+/−0.03)	0.86(+/−0.03)	0.93(+/−0.05)	0.89(+/−0.03)	1145	208	91	1270
Top-4 Features	0.92(+/−0.03)	0.86(+/−0.03)	0.89(+/−0.02)	0.87(+/−0.02)	0.92(+/−0.03)	0.90(+/−0.02)	1166	187	106	1255
Top-5 Features	0.92(+/−0.04)	0.87(+/−0.02)	0.89(+/−0.02)	0.87(+/−0.02)	0.92(+/−0.04)	0.89(+/−0.02)	1173	180	113	1248
Top-6 Features	0.92(+/−0.04)	0.87(+/−0.02)	0.89(+/−0.03)	0.87(+/−0.02)	0.92(+/−0.04)	0.90(+/−0.03)	1172	181	107	1254
Top-7 Features	0.92(+/−0.04)	0.84(+/−0.09)	0.88(+/−0.04)	0.86(+/−0.07)	0.92(+/−0.04)	0.89(+/−0.04)	1139	214	106	1255
Top-8 Features	0.90(+/−0.05)	0.81(+/−0.05)	0.86(+/−0.05)	0.83(+/−0.04)	0.90(+/−0.05)	0.86(+/−0.04)	1095	258	133	1228
Top-9 Features	0.91(+/−0.05)	0.83(+/−0.02)	0.87(+/−0.03)	0.85(+/−0.02)	0.91(+/−0.05)	0.87(+/−0.03)	1129	224	129	1232
Top-10 Features	0.91(+/−0.03)	0.84(+/−0.02)	0.88(+/−0.02)	0.85(+/−0.02)	0.91(+/−0.03)	0.88(+/−0.02)	1141	212	119	1242
Top-11 Features	0.90(+/−0.06)	0.86(+/−0.03)	0.88(+/−0.03)	0.86(+/−0.03)	0.90(+/−0.06)	0.88(+/−0.03)	1157	196	134	1227
Top-12 Features	0.91(+/−0.04)	0.85(+/−0.02)	0.88(+/−0.03)	0.86(+/−0.02)	0.91(+/−0.04)	0.88(+/−0.03)	1153	200	124	1237
Top-13 Features	0.91(+/−0.05)	0.85(+/−0.02)	0.88(+/−0.03)	0.86(+/−0.02)	0.91(+/−0.05)	0.88(+/−0.03)	1148	205	125	1236
Top-14 Features	0.91(+/−0.05)	0.86(+/−0.03)	0.88(+/−0.03)	0.86(+/−0.03)	0.91(+/−0.05)	0.88(+/−0.03)	1157	196	128	1233
Top-15 Features	0.89(+/−0.02)	0.84(+/−0.03)	0.87(+/−0.02)	0.85(+/−0.02)	0.89(+/−0.02)	0.87(+/−0.02)	1139	214	148	1213
Top-16 Features	0.91(+/−0.05)	0.86(+/−0.03)	0.89(+/−0.04)	0.87(+/−0.03)	0.91(+/−0.05)	0.89(+/−0.04)	1167	186	120	1241
Top-17 Features	0.91(±0.04)	0.86(±0.04)	0.89(±0.04)	0.87(±0.03)	0.91(±0.04)	0.89(±0.04)	1165	189	116	1245

Abbreviations: TN, true negative; FP, false positive; FN, false negative; TP, true positive

Supplementary Table S2. Performance analysis of LR models using top 1 to top 17 features from ERT feature ranking technique.

	Sensitivity	Specificity	Accuracy	Precision	Recall	F1 Score	Non-CKD		CKD	
							TN	FP	FN	TP
Top-1 Feature	0.95(+/−0.02)	0.72(+/−0.03)	0.83(+/−0.02)	0.77(+/−0.02)	0.95(+/−0.02)	0.85(+/−0.01)	974	379	72	1289
Top-2 Features	0.93(+/−0.04)	0.84(+/−0.03)	0.88(+/−0.03)	0.85(+/−0.02)	0.93(+/−0.04)	0.89(+/−0.03)	1134	219	101	1260
Top-3 Features	0.92(+/−0.01)	0.84(+/−0.03)	0.88(+/−0.02)	0.85(+/−0.02)	0.92(+/−0.01)	0.89(+/−0.02)	1131	222	104	1257
Top-4 Features	0.92(+/−0.05)	0.86(+/−0.01)	0.89(+/−0.02)	0.87(+/−0.01)	0.92(+/−0.05)	0.89(+/−0.02)	1167	186	113	1248
Top-5 Features	0.91(+/−0.04)	0.86(+/−0.00)	0.88(+/−0.02)	0.86(+/−0.01)	0.91(+/−0.04)	0.89(+/−0.02)	1158	195	122	1239
Top-6 Features	0.92(+/−0.04)	0.87(+/−0.02)	0.89(+/−0.03)	0.87(+/−0.02)	0.92(+/−0.04)	0.90(+/−0.03)	1172	181	108	1253
Top-7 Features	0.93(+/−0.03)	0.86(+/−0.01)	0.89(+/−0.02)	0.87(+/−0.01)	0.93(+/−0.03)	0.90(+/−0.02)	1167	186	99	1262
Top-8 Features	0.92(+/−0.05)	0.84(+/−0.04)	0.88(+/−0.03)	0.85(+/−0.03)	0.92(+/−0.05)	0.89(+/−0.03)	1136	217	103	1258
Top-9 Features	0.91(+/−0.04)	0.84(+/−0.02)	0.88(+/−0.02)	0.85(+/−0.02)	0.91(+/−0.04)	0.88(+/−0.02)	1142	211	118	1243
Top-10 Features	0.91(+/−0.04)	0.84(+/−0.03)	0.87(+/−0.03)	0.85(+/−0.03)	0.91(+/−0.04)	0.88(+/−0.03)	1137	216	125	1236
Top-11 Features	0.91(+/−0.01)	0.85(+/−0.02)	0.88(+/−0.01)	0.86(+/−0.02)	0.91(+/−0.01)	0.88(+/−0.01)	1153	200	126	1235
Top-12 Features	0.91(+/−0.02)	0.85(+/−0.03)	0.88(+/−0.02)	0.86(+/−0.03)	0.91(+/−0.02)	0.88(+/−0.02)	1146	207	121	1240
Top-13 Features	0.90(+/−0.04)	0.86(+/−0.04)	0.88(+/−0.04)	0.86(+/−0.03)	0.90(+/−0.04)	0.88(+/−0.03)	1158	195	130	1231
Top-14 Features	0.90(+/−0.05)	0.86(+/−0.04)	0.88(+/−0.04)	0.86(+/−0.04)	0.90(+/−0.05)	0.88(+/−0.04)	1159	194	132	1229
Top-15 Features	0.89(+/−0.02)	0.84(+/−0.03)	0.86(+/−0.02)	0.85(+/−0.02)	0.89(+/−0.02)	0.87(+/−0.02)	1138	215	152	1209
Top-16 Features	0.91(+/−0.04)	0.86(+/−0.04)	0.89(+/−0.04)	0.87(+/−0.04)	0.91(+/−0.04)	0.89(+/−0.03)	1162	191	119	1242
Top-17 Features	0.89(+/−0.07)	0.85(+/−0.03)	0.87(+/−0.05)	0.86(+/−0.03)	0.89(+/−0.07)	0.87(+/−0.05)	1148	205	146	1215
